# Supplementary material for: Highly stacked 3D organic integrated circuits with via-hole-less multilevel metal interconnects
Source: Nat Commun. 2019 Jun 3;10:2424. doi: 10.1038/s41467-019-10412-9 (PMC6546689; doi:10.1038/s41467-019-10412-9)
Supplement: Supplementary file 1 — Supplementary Information [file 41467_2019_10412_MOESM1_ESM.pdf]

Supplementary Information

Highly Stacked 3D Organic Integrated Circuits with Via-Hole-Less Multilevel Metal Interconnects

Yoo *et al.*

Supplementary Figures

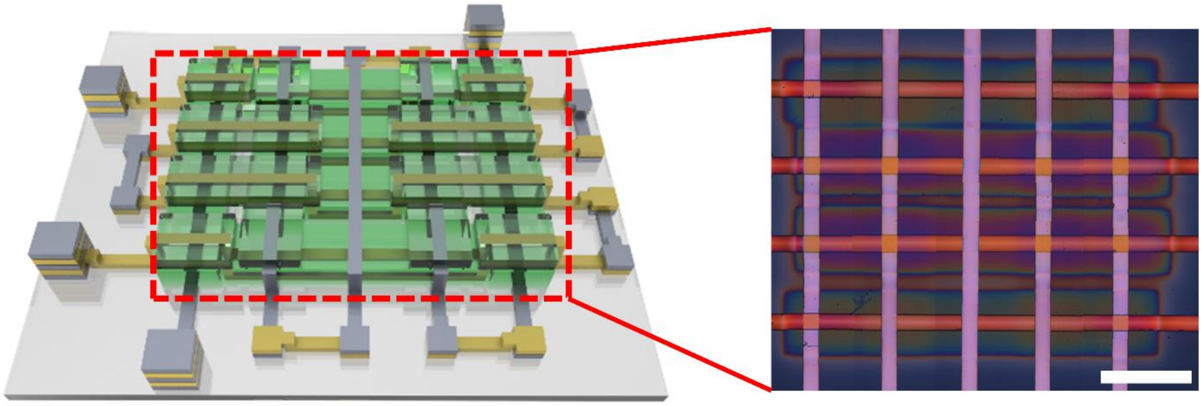

Supplementary Figure 1 | Multilevel metal interconnect test structure. To observe the patterned PV3D3 layers, we reproduced a multilevel metal interconnect test structure on a SiO_2/Si substrate because PV3D3 layers are transparent on glass substrate. Scale bar, 1,400 μm.

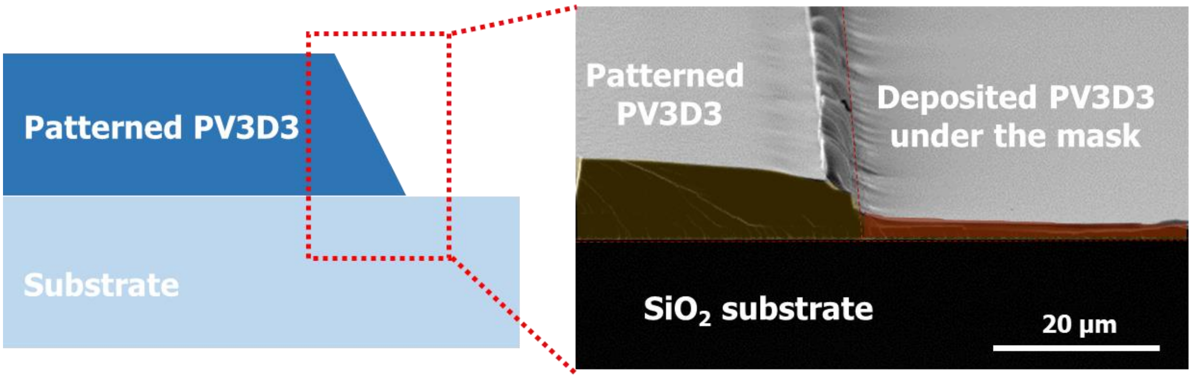

Supplementary Figure 2 | False-colored cross-section SEM image. Patterned PV3D3 (yellow region) and undesired deposition of PV3D3 under the shadow mask (red region).

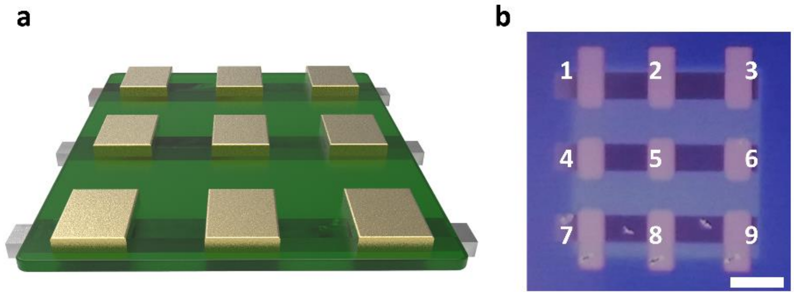

c

44.65 nF/cm^2	45.20 nF/cm^2	46.54 nF/cm^2
44.18 nF/cm^2	44.69 nF/cm^2	46.14 nF/cm^2
39.52 nF/cm^2	44.79 nF/cm^2	46.31 nF/cm^2

Supplementary Figure 3 | Capacitance values of the patterned PV3D3 layer. **a,** Schematic of the MIM capacitor based on pattern PV3D3. **b,** Optical microscopy image of the MIM capacitor. Scale bar, 500 μm. **c,** Measured capacitance values. The measured capacitance values were normalized to the unit area in cm^2.

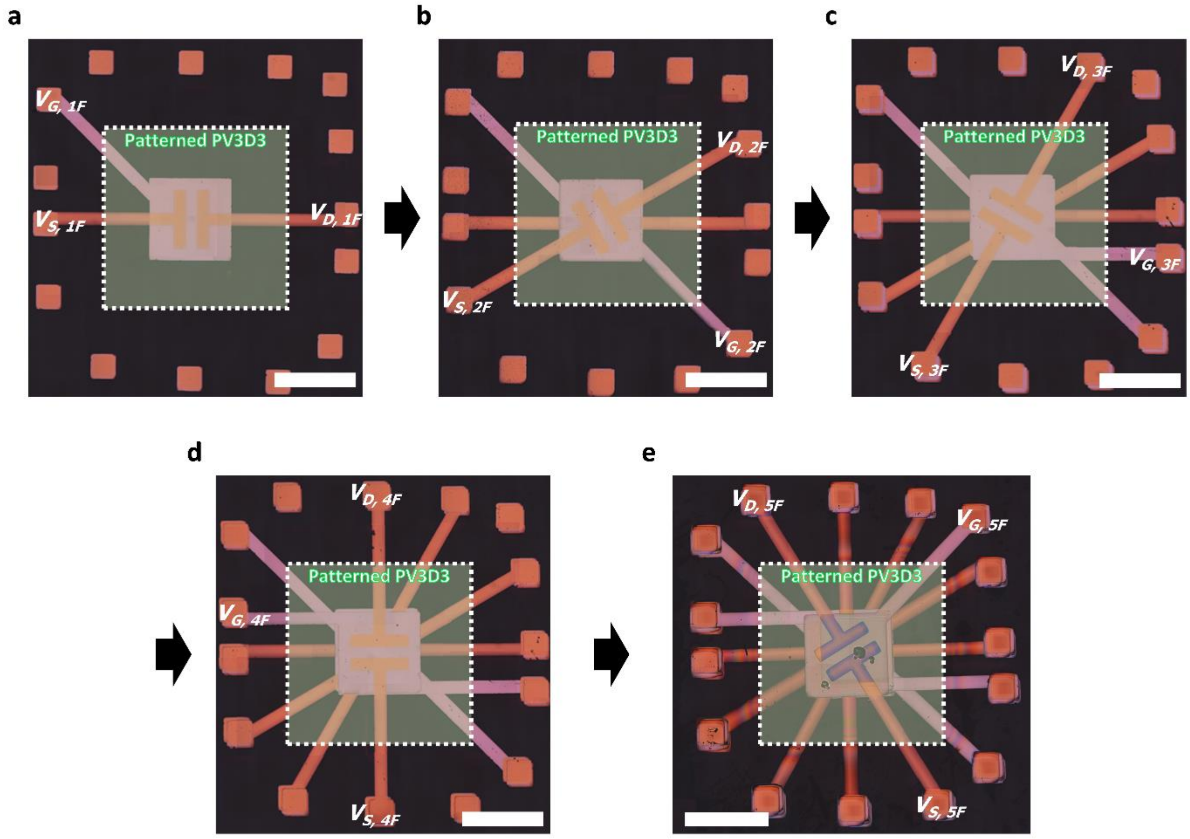

Supplementary Figure 4 | Process flow of 3D-organic thin-film transistors. **a,** Gate, source, and drain electrodes on the 1st floor. Scale bar, 1,200 μm. **b,** Gate, source, and drain electrodes on the 2nd floor. Scale bar, 1,200 μm. **c,** Gate, source, and drain electrodes on the 3rd floor. Scale bar, 1,200 μm. **d,** Gate, source, and drain electrodes on the 4th floor. Scale bar, 1,200 μm. **e,** Completed 3D-OTFT. Scale bar, 1,200 μm.

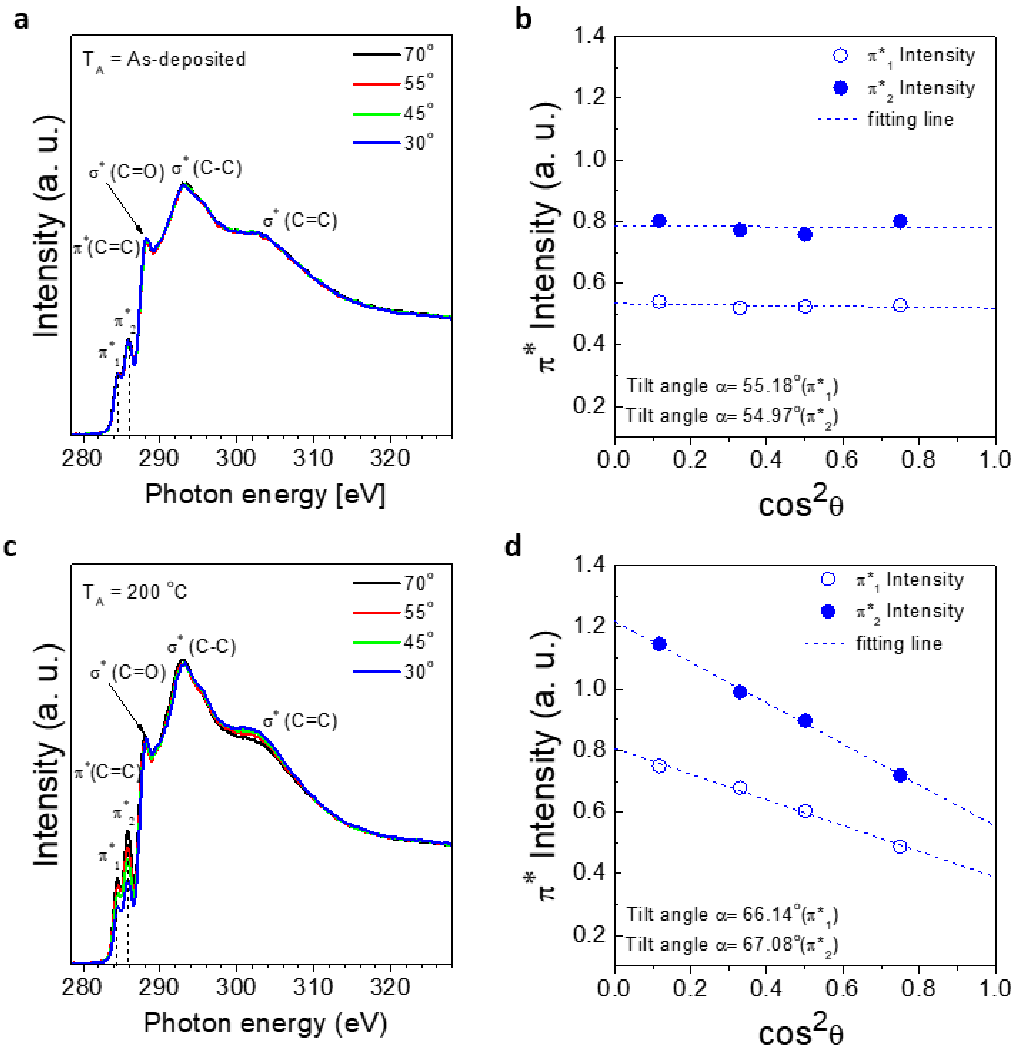

Supplementary Figure 5 | Annealing effect on PTCDI-C13. a, Near-edge X-ray absorption of fine structure (NEXAFS) spectra of *N,N'*-ditridecylperylenediimide (PTCDI-C13) without annealing. **b,** Intensities of π* transitions versus incidence angle. Using NEXAFS spectroscopy, the average orientation of the PTCDI-C13 π-conjugated planes was investigated. The tilting angle (α) is ~55.18°, which indicates the random orientation of the molecular structure. **c,** NEXAFS spectra for PTCDI-C13 at the annealing temperature (T_A) = 200 °C. **d,** Intensities of π* transitions versus incidence angle. The tilting angle (α) is ~67.08°, which indicates the high edge-on orientation of the molecular structure. NEXAFS experiments were conducted at the 4D beamline of the Pohang Accelerator Laboratory (PAL). NEXAFS experiments were conducted under ultrahigh vacuum (<10^{-9} torr). The partial electron yield (PEY) mode was used.

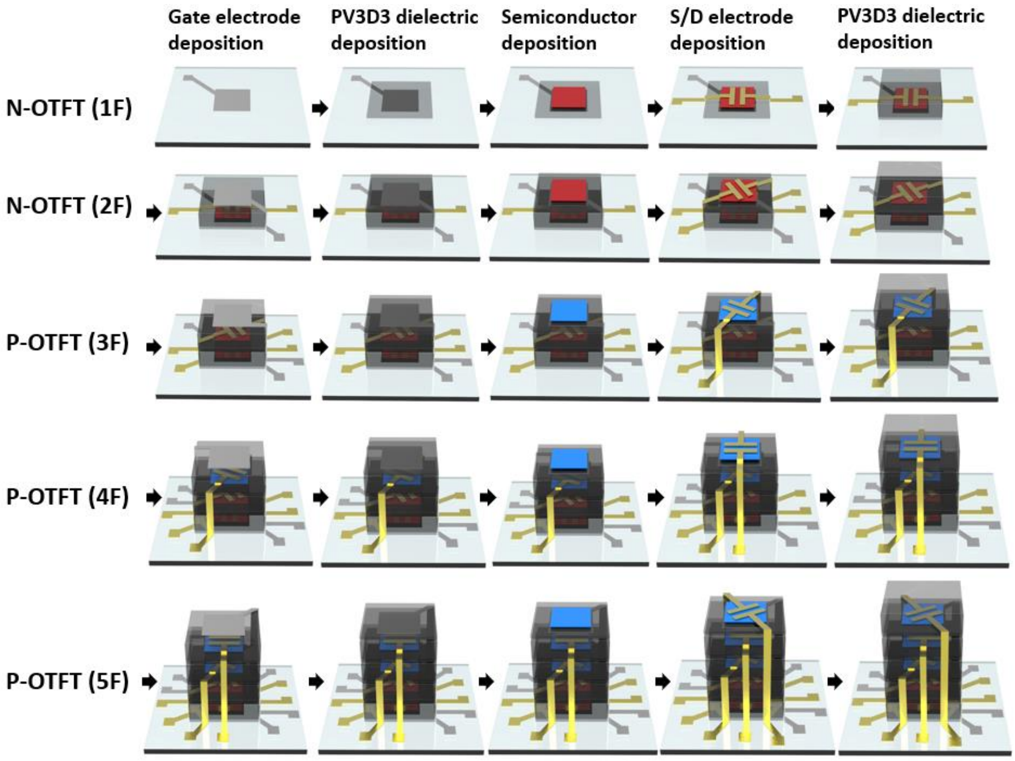

Supplementary Figure 6 | Schematic of fabrication process flow. Vertcial stacking of 5 OTFTs.

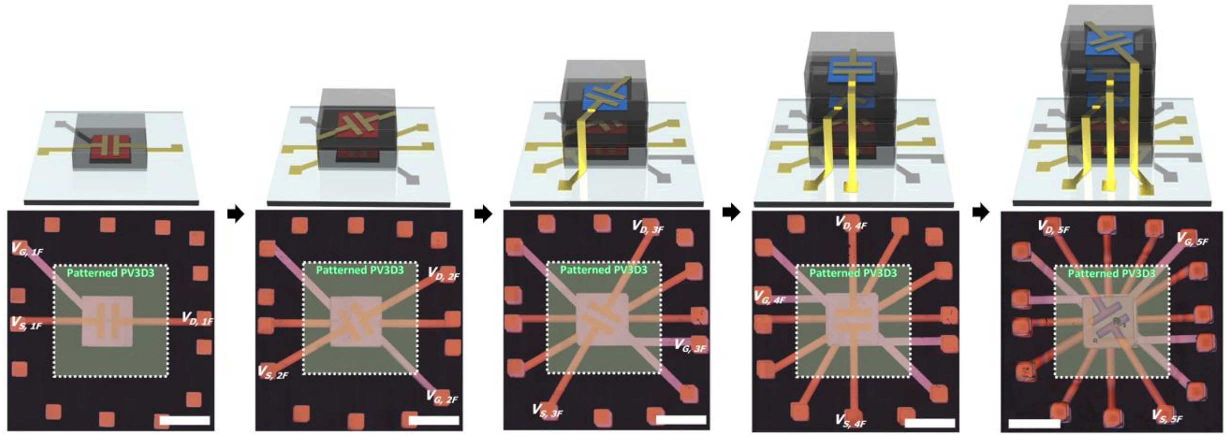

Supplementary Figure 7 | Schematic and optical images of 3D-OTFTs. Scale bar, 1,400 μm

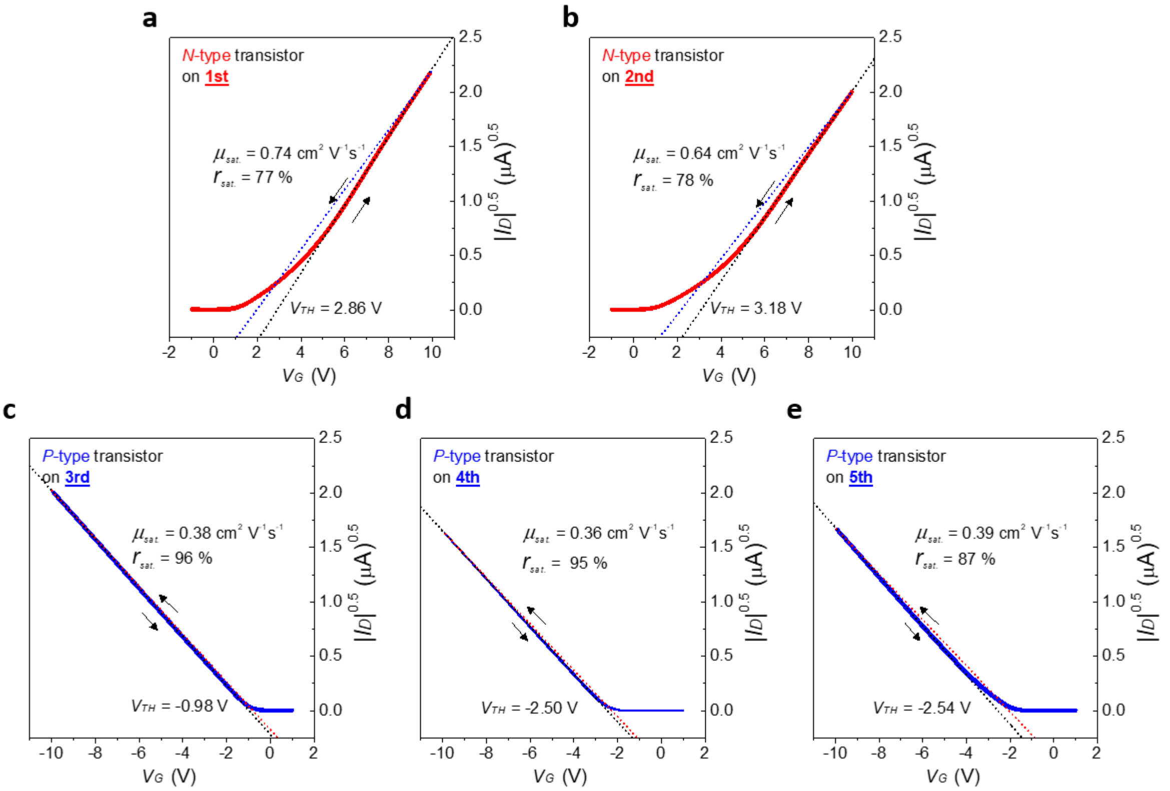

Supplementary Figure 8 | Reliability factor in the saturation region. a-b, Transfer curves of PTCDI-C13-based n-type OTFTs on the 1st and 2nd floors of a 3D-stacked OTFT. V_D = 10 V. **c-e,** Transfer curves of DNTT-based p-type OTFTs on the 3rd, 4th, and 5th floors of a 3D-stacked OTFT. V_D = -10 V.

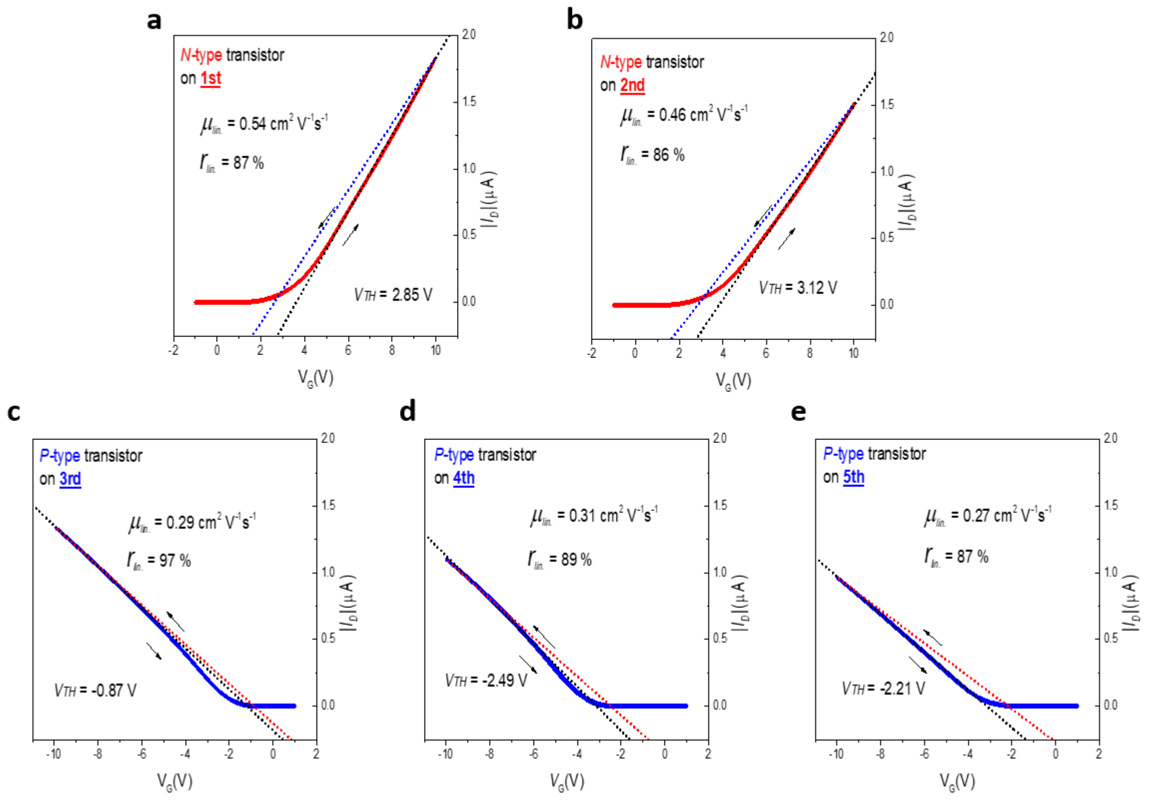

Supplementary Figure 9 | Reliability factor in the linear region. a-b, Transfer curves of PTCDI-C13-based n-type OTFTs on the 1st and 2nd floors of a 3D-stacked OTFT. V_D = 2 V. **c-e,** Transfer curves of DNTT-based p-type OTFTs on the 3rd, 4th, and 5th floors of a 3D-stacked OTFT. V_D = -2 V.

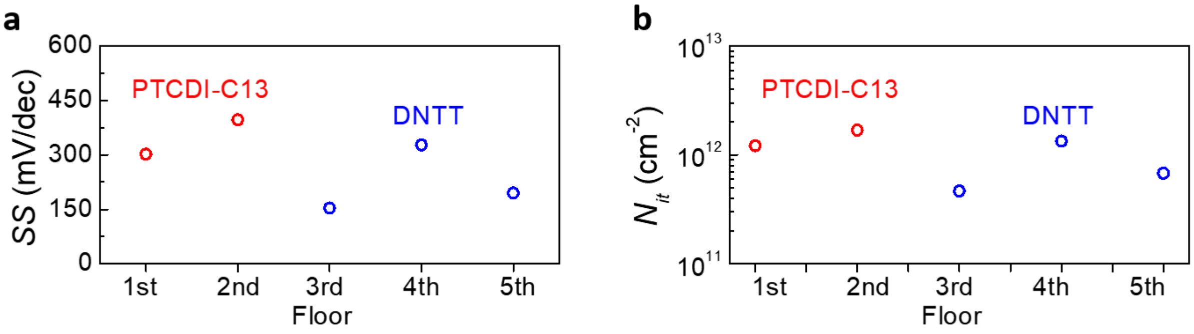

Supplementary Figure 10 | Interfacial traps in 3D-stacked OTFTs. a, Extracted subthreshold swing values of 3D-stacked OTFTs. **b,** Extracted interfacial trap density values of 3D-stacked OTFTs.

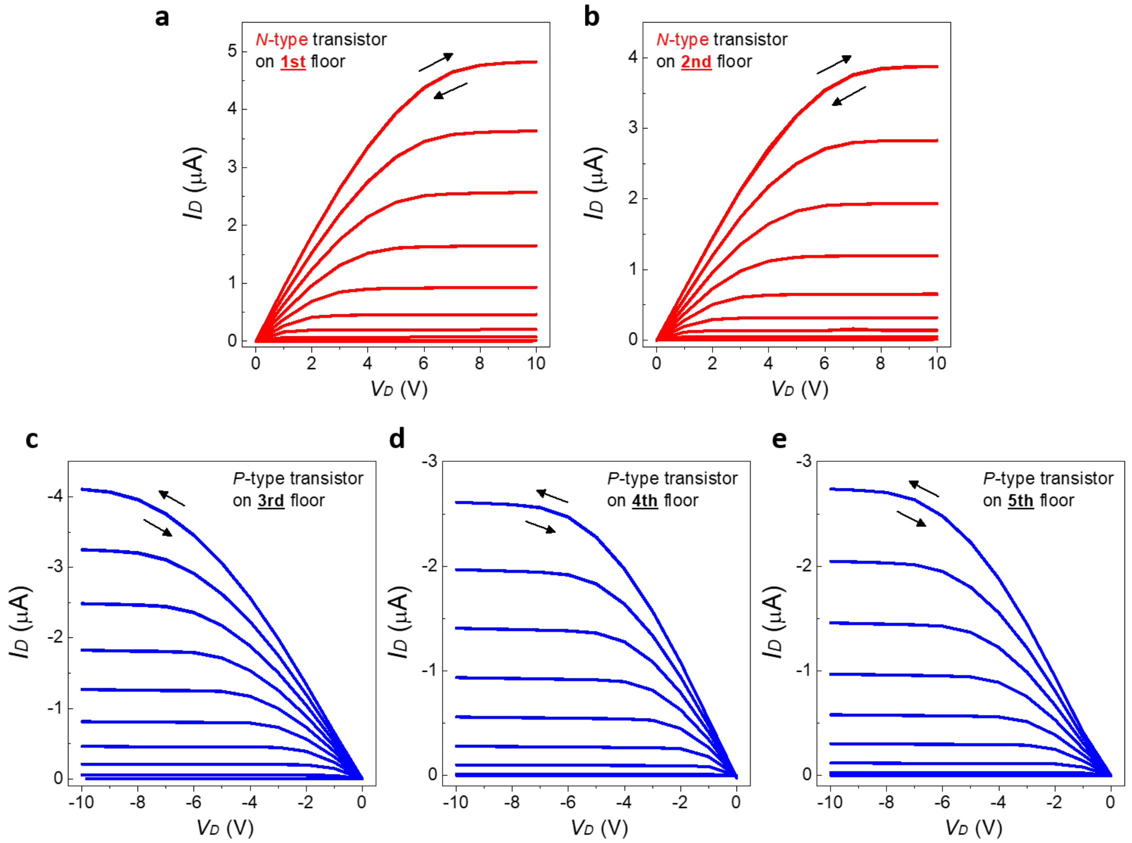

Supplementary Figure 11 | Output characteristics of 3D-stacked OTFTs. a-b, Output curves of PTCDI-C13-based n-type OTFTs on the 1st and 2nd floors of a 3D-stacked OTFT. V_G = 0~10 V (step = 1 V). **c-e,** Transfer curves of DNTT-based p-type OTFTs on the 3rd, 4th, and 5th floors of a 3D-stacked OTFT. V_G = 0~-10 V (step = -1 V).

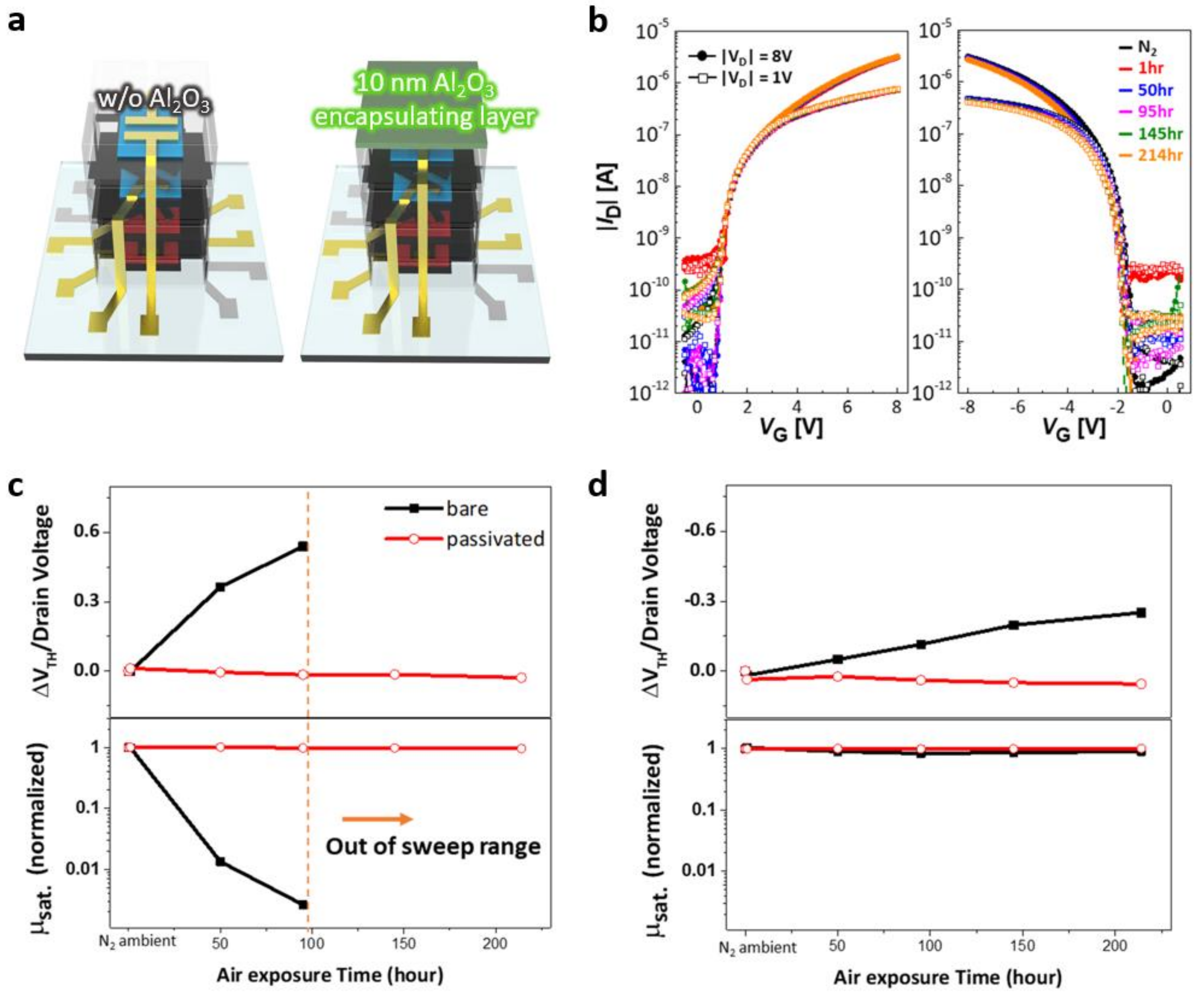

Supplementary Figure 12 | Air-stability of 3D-stacked OTFTs. a, Schematic structures of the Al_2O_3 encapsulated 3D-stacked OTFTs **b,** Transfer curves of p-type and n-type 3D-stacked OTFTs with Al_2O_3 encapsulation. **c,** Plot of mobility and V_{TH} as a function of the air-exposure time for the n-type 3D-stacked OTFT. **d,** Plot of mobility and V_{TH} as a function of the air-exposure time for the p-type 3D-stacked OTFT.

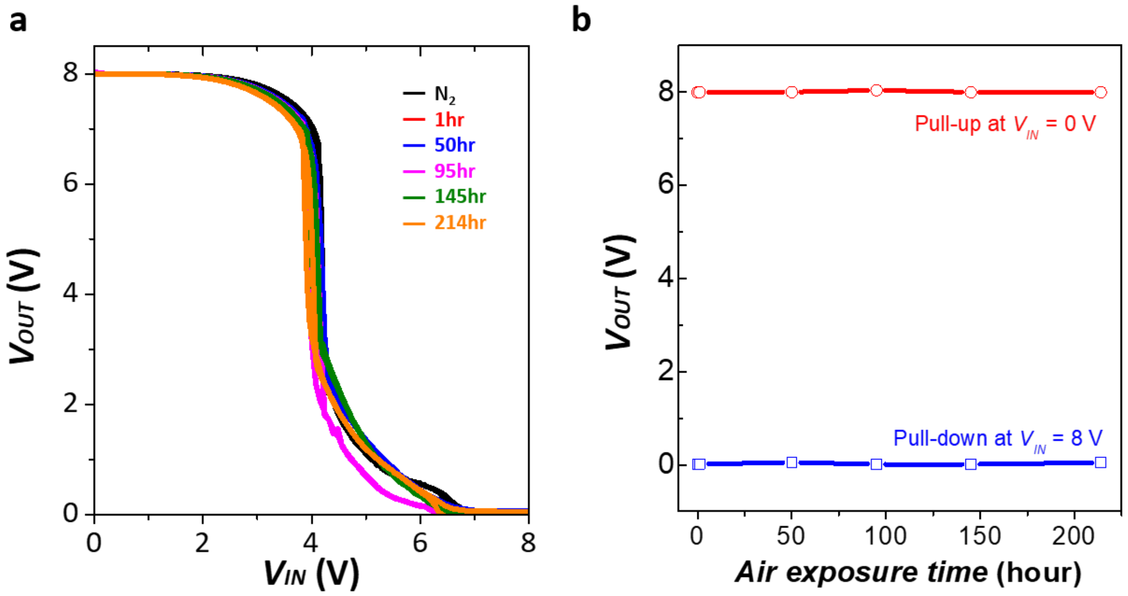

Supplementary Figure 13 | Air-stability of the Al_2O_3 encapsulated 3D-stacked inverter circuit. a, Inverter transfer characteristics as a function of the air-exposure time for the Al_2O_3 encapsulated 3D-stacked inverter circuit **b,** Plot of pull-up and pull-down V_{OUT} values as a function of the air-exposure time.

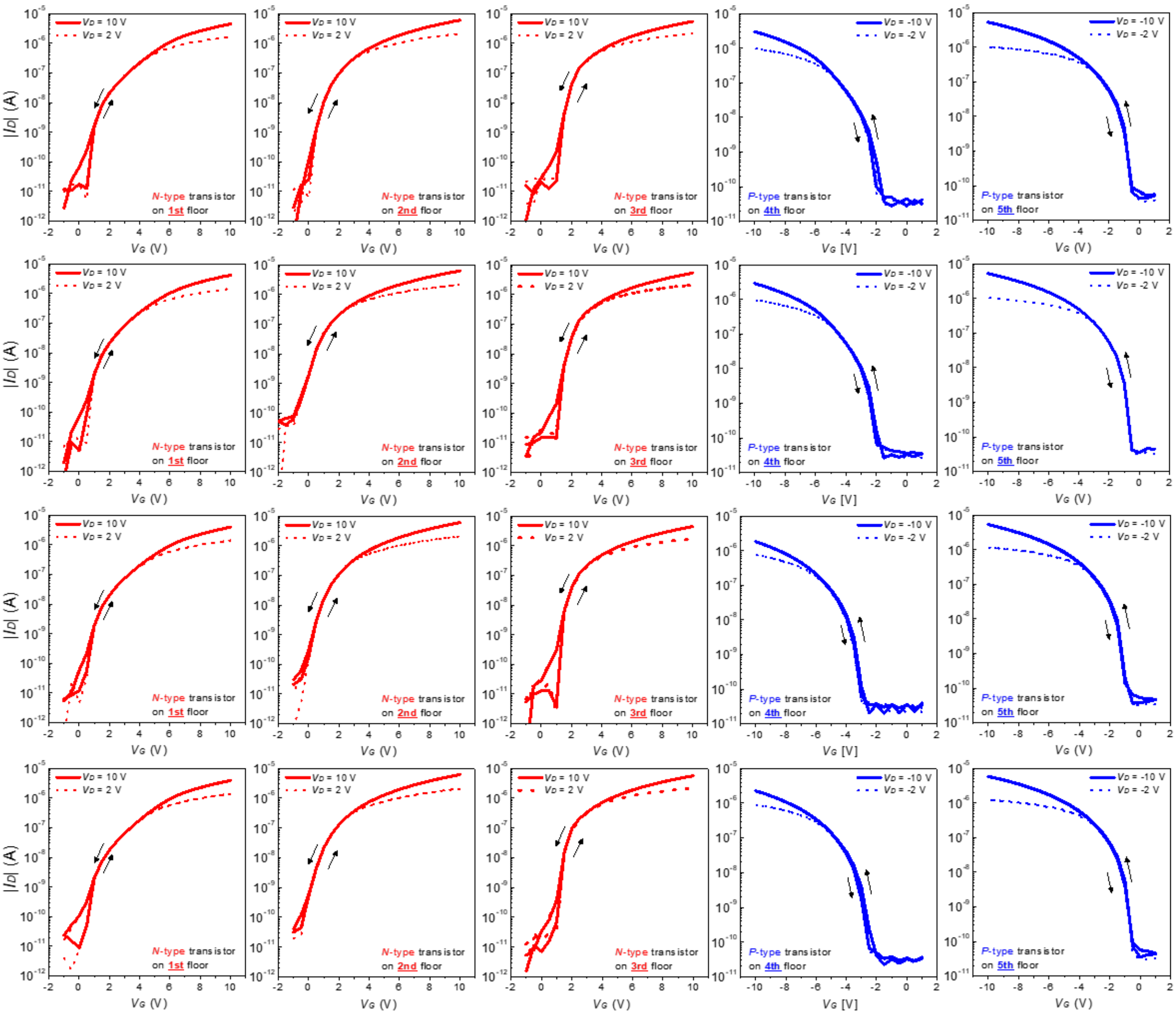

Supplementary Figure 14 | Transfer characteristics of 20 OTFTs.

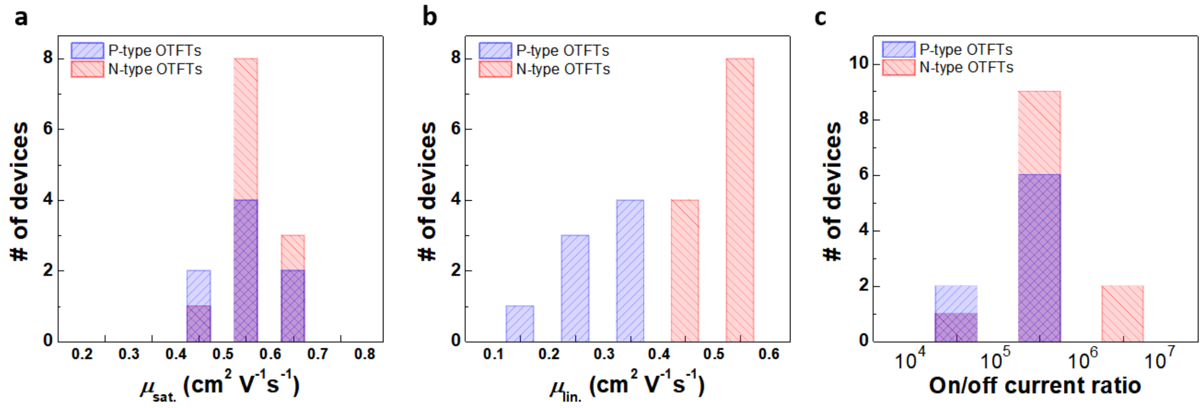

Supplementary Figure 15 | Reproducibility and uniformity results. a, Histograms of carrier mobility of 20 OTFTs under saturation region (V_D = 10 V). **b,** Histograms of carrier mobility of 20 OTFTs under linear region (V_D = 2V). **c,** Histograms of on/off current ratio of 20 OTFTs.

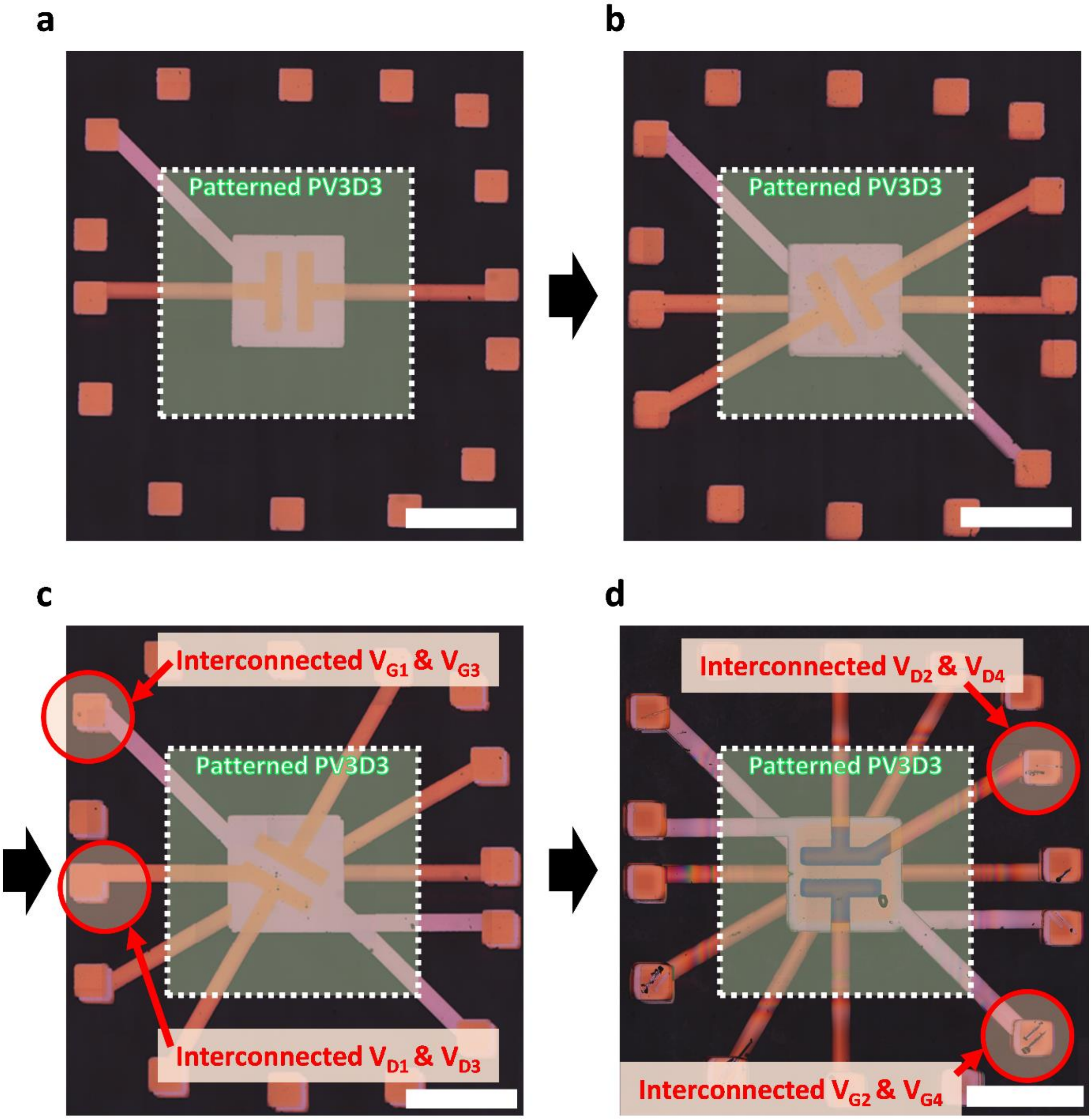

Supplementary Figure 16 | Process flow of 3D-inverters. a, Gate, source, and drain electrodes on the 1st floor. Scale bar, 1,200 μm. **b,** Gate, source, and drain electrodes on the 2nd floor. Scale bar, 1,200 μm. **c,** Gate, source, and drain electrodes on the 3rd floor. Gate electrodes on the 1st and 3rd layers were interconnected. Drain electrodes on the 1st and 3rd layers were interconnected. Scale bar, 1,200 μm. **d,** Completed 3D-integrated inverters. Gate electrodes on the 2nd and 4th layers were interconnected. Drain electrodes on the 2nd and 4th layers were interconnected. Scale bar, 1,200 μm.

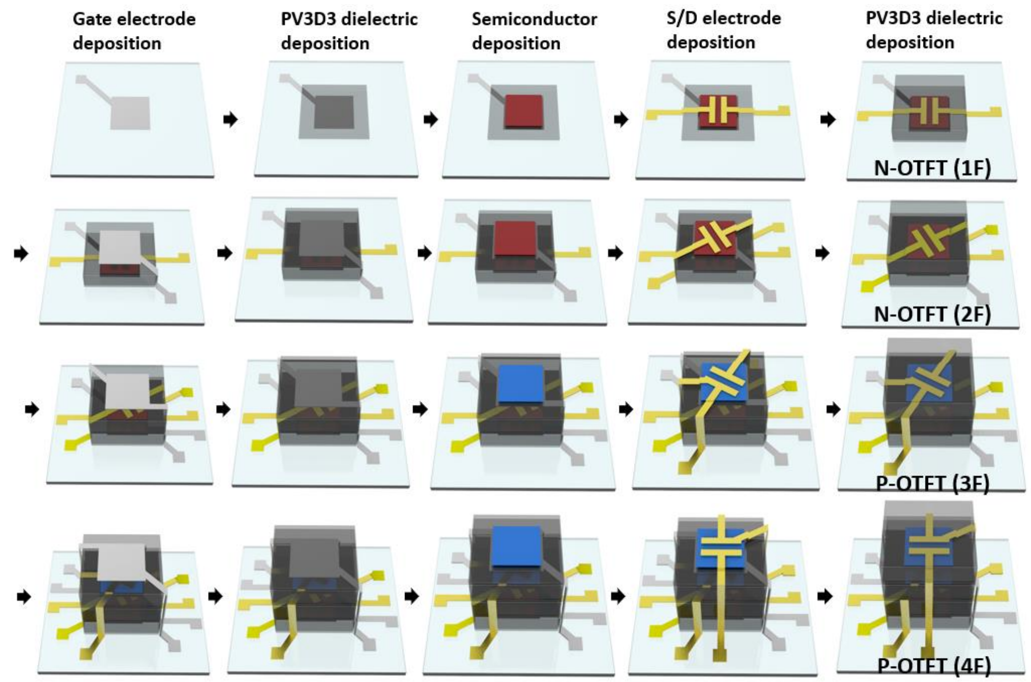

Supplementary Figure 17 | Process flow of 3D-stacked inverter circuits.

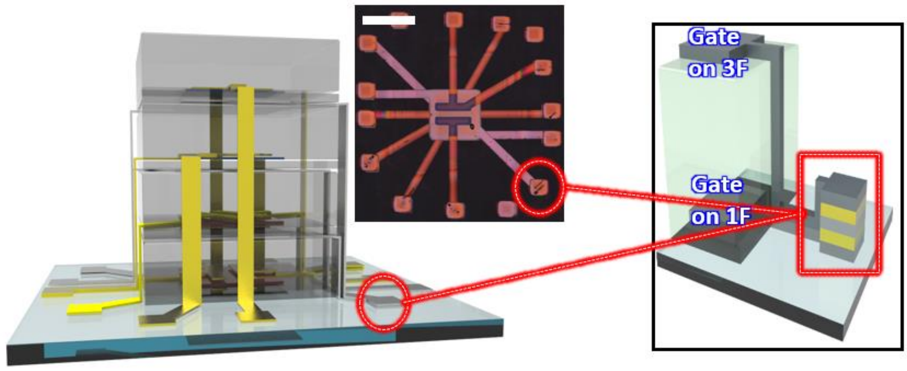

Supplementary Figure 18 | Schematic of the gate metal interconnection.
Scale bar, 1,400 μm

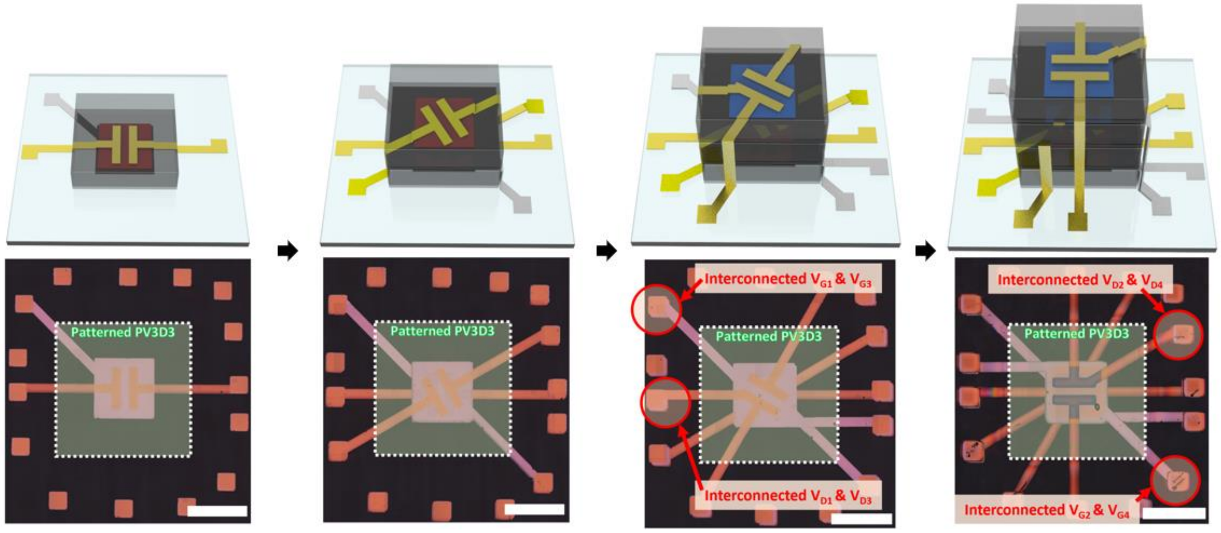

Supplementary Figure 19 | Process flow of 3D-inverters. Scale bar, 1,400 μm. For the 3D-stacked inverter circuits, two complementary inverter circuits were fabricated, consisting of four OTFTs: two n-type OTFTs on 1F-2F and two p-type OTFTs on 3F-4F (Supplementary Figure 23). The OTFT was surrounded by an open area through which the electrodes were connected vertically to each other. Supplementary Figure 24 shows an example of the metal interconnection of the gate electrode on 1F and the other gate electrode on 3F. The gate electrodes on 2F and 4F, the drain electrodes on 1F and 3F, and the drain electrodes on 2F and 4F were respectively interconnected in the same manner.

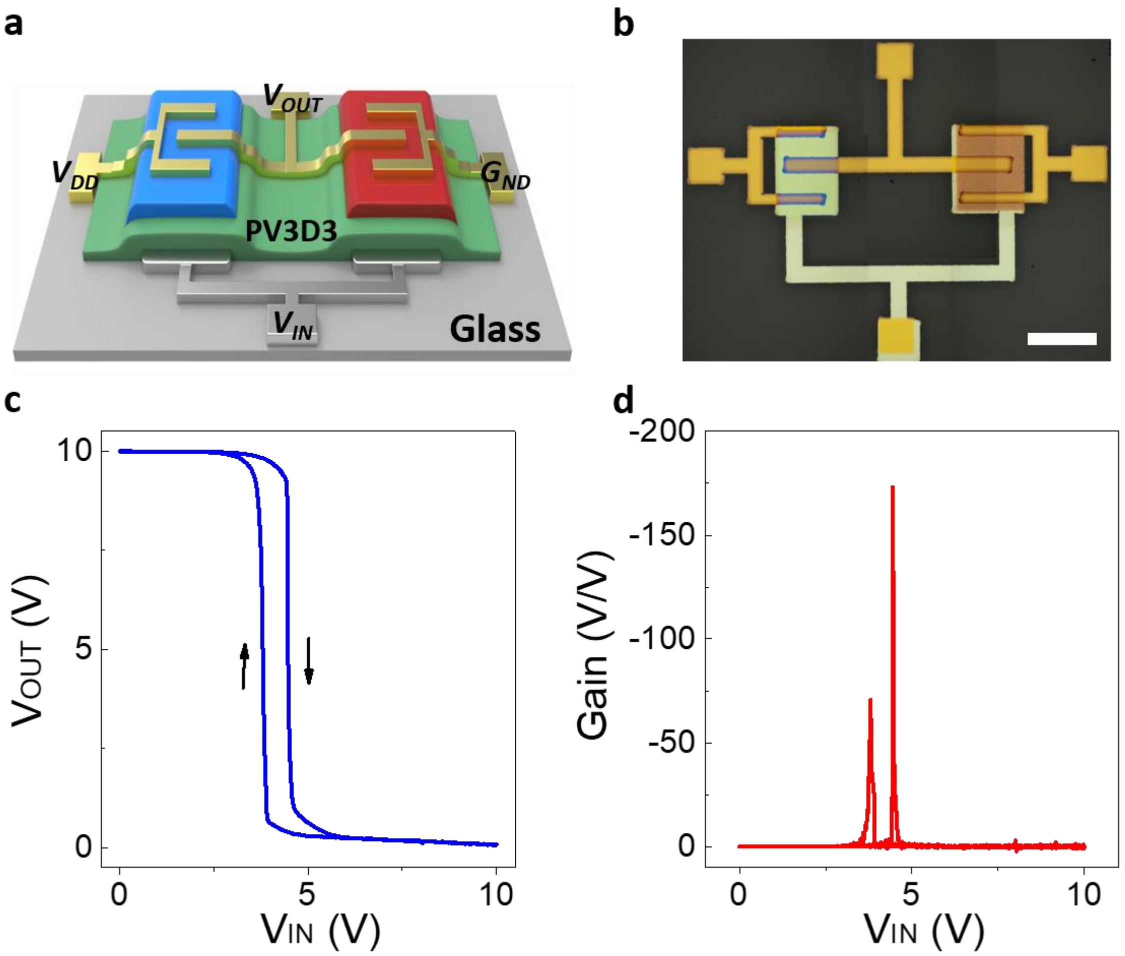

Supplementary Figure 20 | Conventional OTFTs inverter circuit. a, Schematic of a conventional CMOS inverter circuit. **b,** Optical microscopy image of the fabricated conventional CMOS inverter circuit. (scale bar = 700 μm) **c,** Transfer characteristics of the fabricated conventional CMOS inverter circuit at V_{DD} = 10 V. **d,** DC gain values as a function of applied V_{IN}.

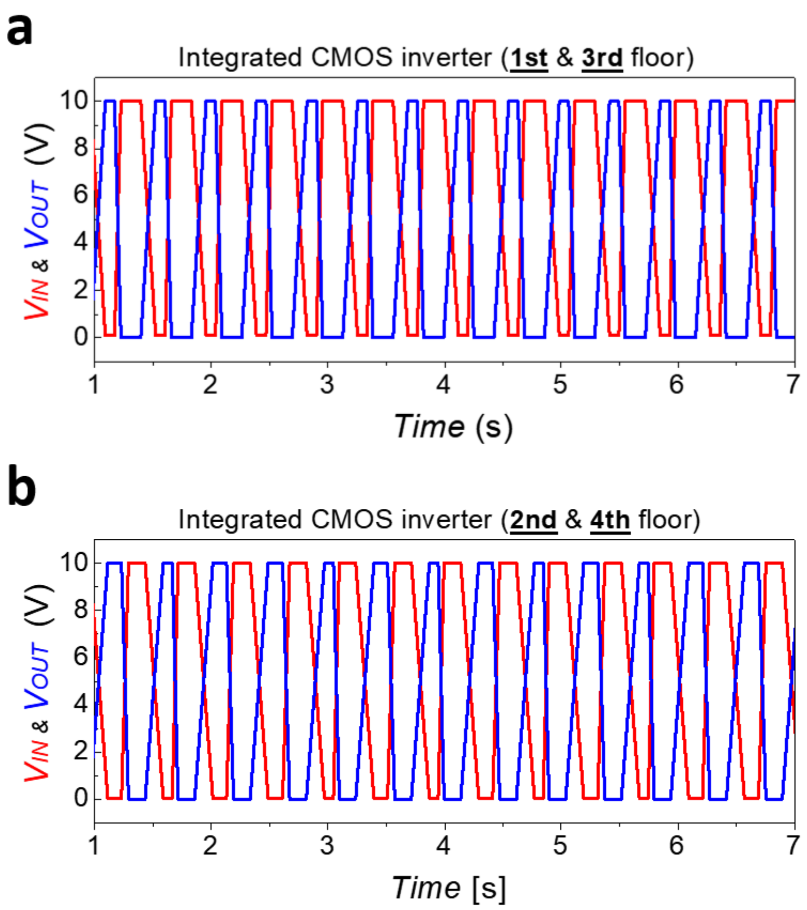

Supplementary Figure 21 | Transient measurements for inverter circuits. a, Measured output voltage values as a function of the input voltage pulse for inverter circuit integrated transistors on the 1st and 3rd floors. **b,** Measured output voltage values as a function of the input voltage pulse for inverter circuit integrated transistors on the 2nd and 4th floors.

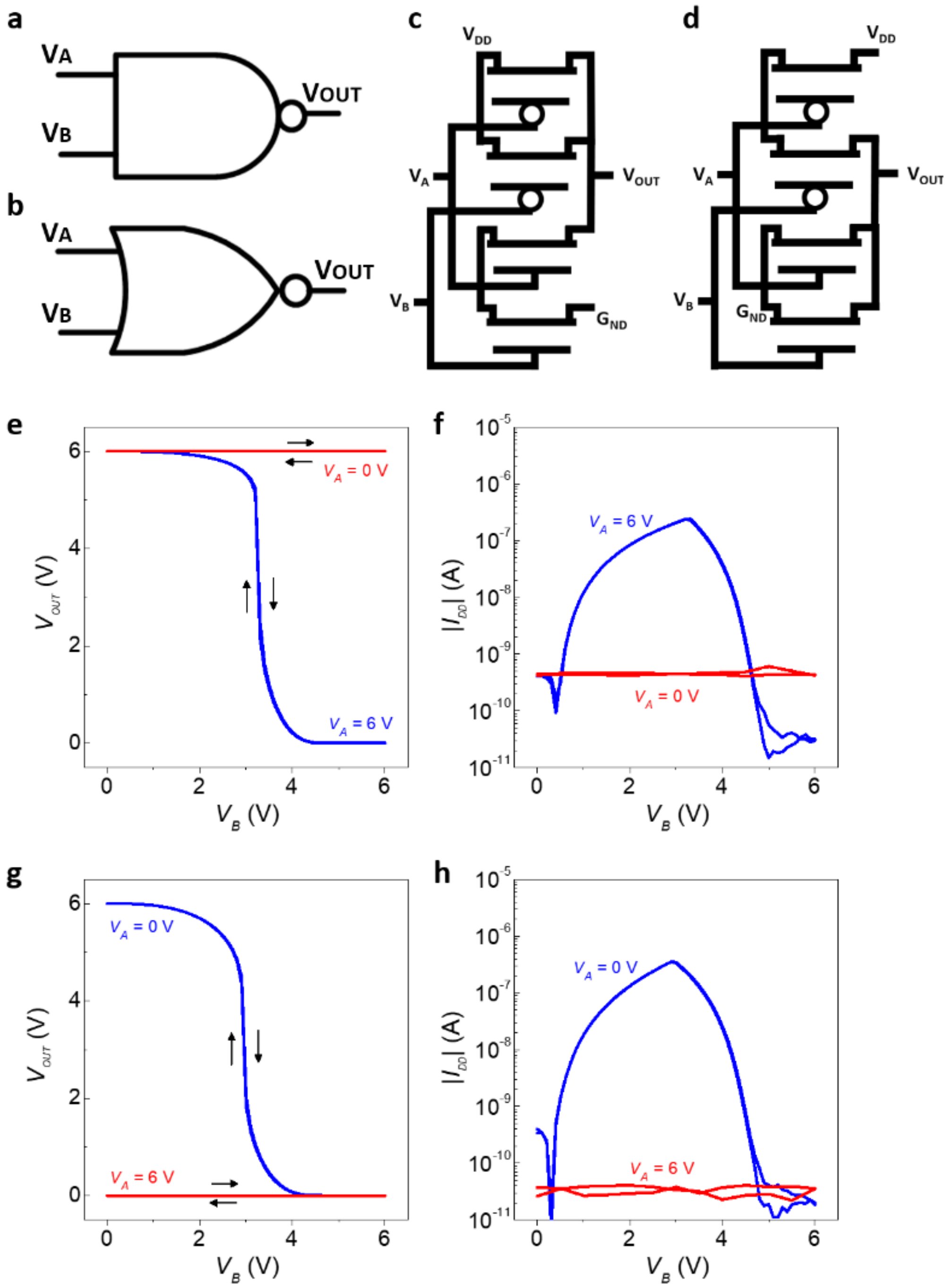

Supplementary Figure 22 | 3D-stacked NAND and NOR logic circuits. a, Symbol of NAND circuit. **b,** Symbol of NOR circuit. **c,** Schematic of 3D-stacked NAND circuit. **d,** Schematic of 3D-stacked NOR circuit. **e,** DC transfer characteristics of NAND circuit **f,** The corresponding I_{DD} plot of NAND circuit. **g,** DC transfer characteristics of NOR circuit **h,** The corresponding I_{DD} plot of NOR circuit.

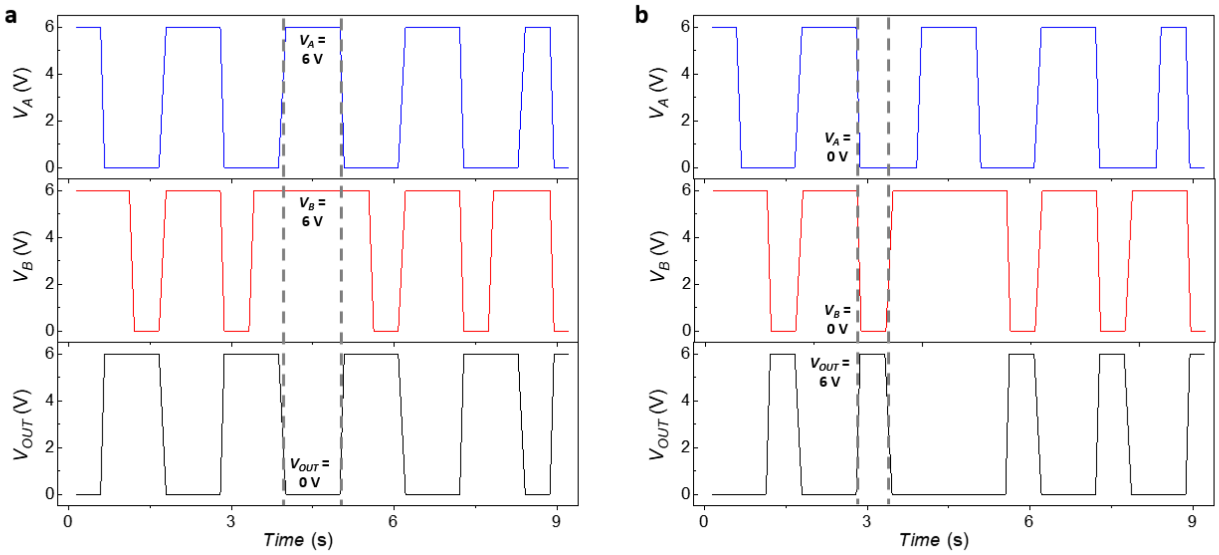

Supplementary Figure 23 | Transient measurement of NAND and NOR. a, Measured transient result of NAND circuit with respect to V_A and V_B. **b,** Measured transient result of NOR circuit with respect to V_A and V_B.

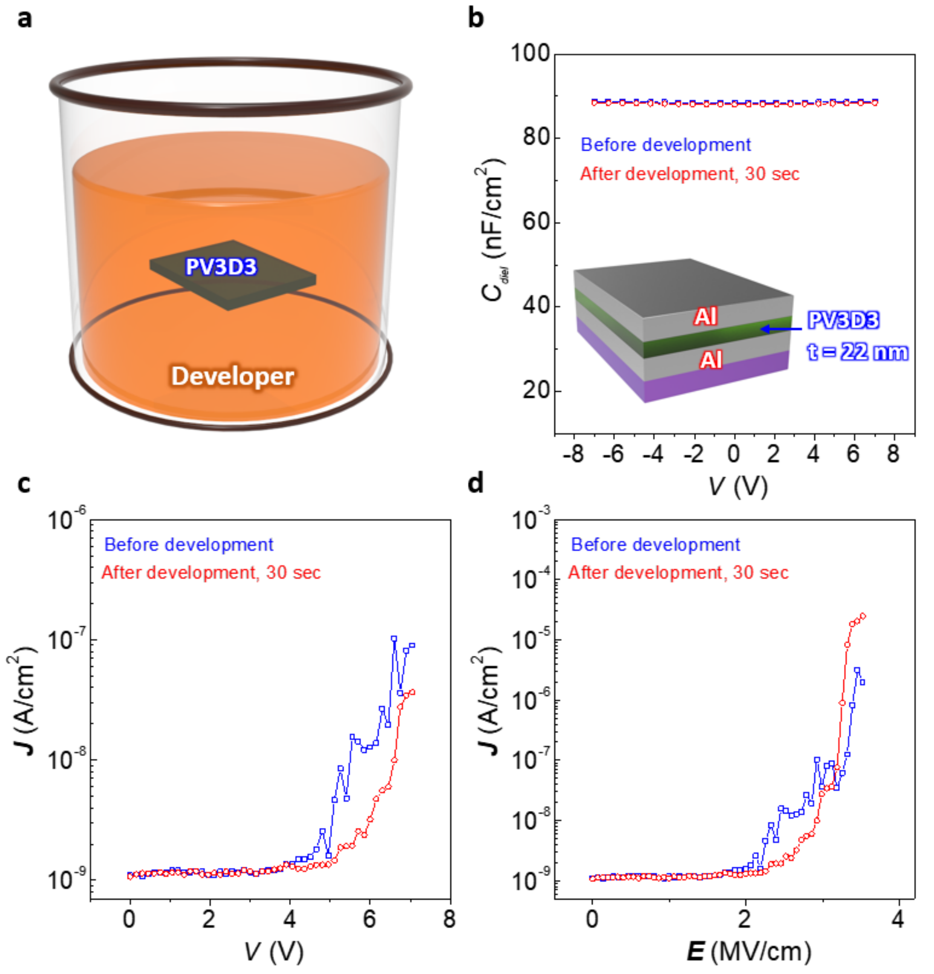

Supplementary Figure 24 | Development effect on PV3D3. a, Schematic illustration of dipping PV3D3 in developer solvent. **b**, C_{diel}-V characteristics of 22 nm PV3D3 dielectric layer between Al electrodes before/after development process. **c**, leakage current densities (J)-applied voltage (V) measured for 22 nm PV3D3 before/after development process. **d**, leakage current densities (J)-applied electric field (E) measured for 22 nm PV3D3 before/after development process.

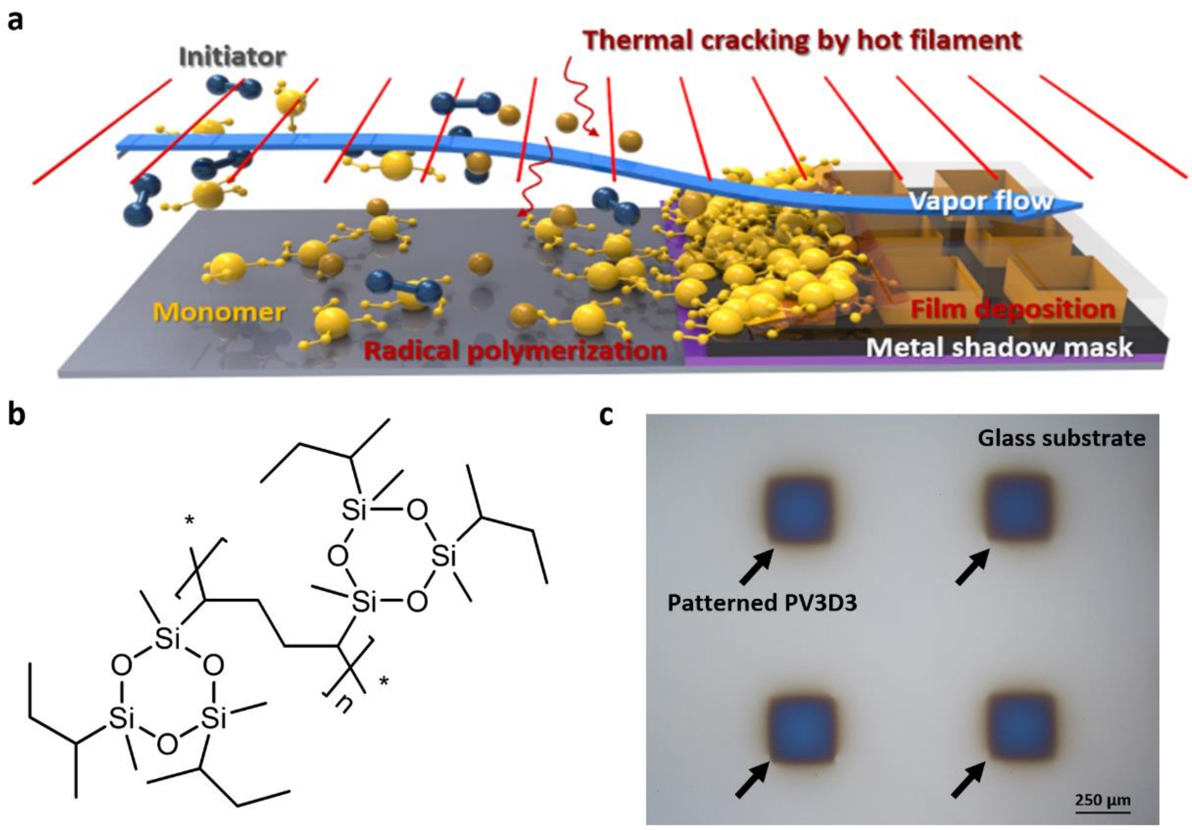

Supplementary Figure 25 | Patterned PV3D3 deposition process by iCVD. a, Schematic illustration of patterned poly(1,3,5-trimethyl-1,3,5-trivinyl cyclotrisiloxane) (PV3D3) deposition. **b,** Molecular structure of PV3D3. **c,** Optical micrograph image of deposited PV3D3.

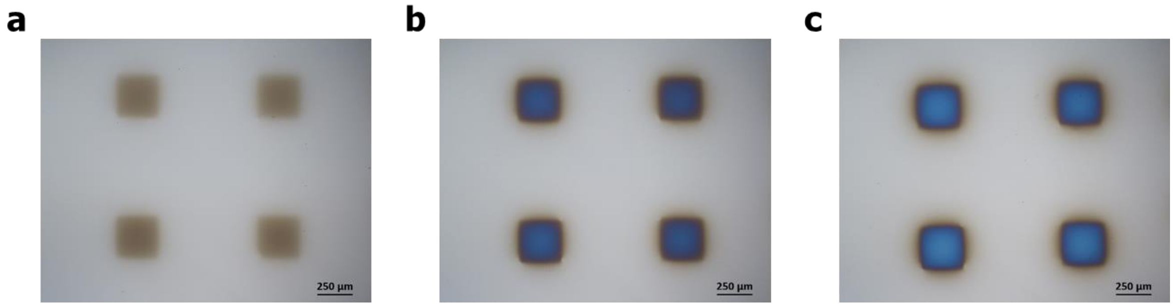

Supplementary Figure 26 | Optical microscopy images of patterned PV3D3.
a, thickness of ~30 nm. **b,** thickness of ~80 nm. **c,** thickness of ~130 nm.

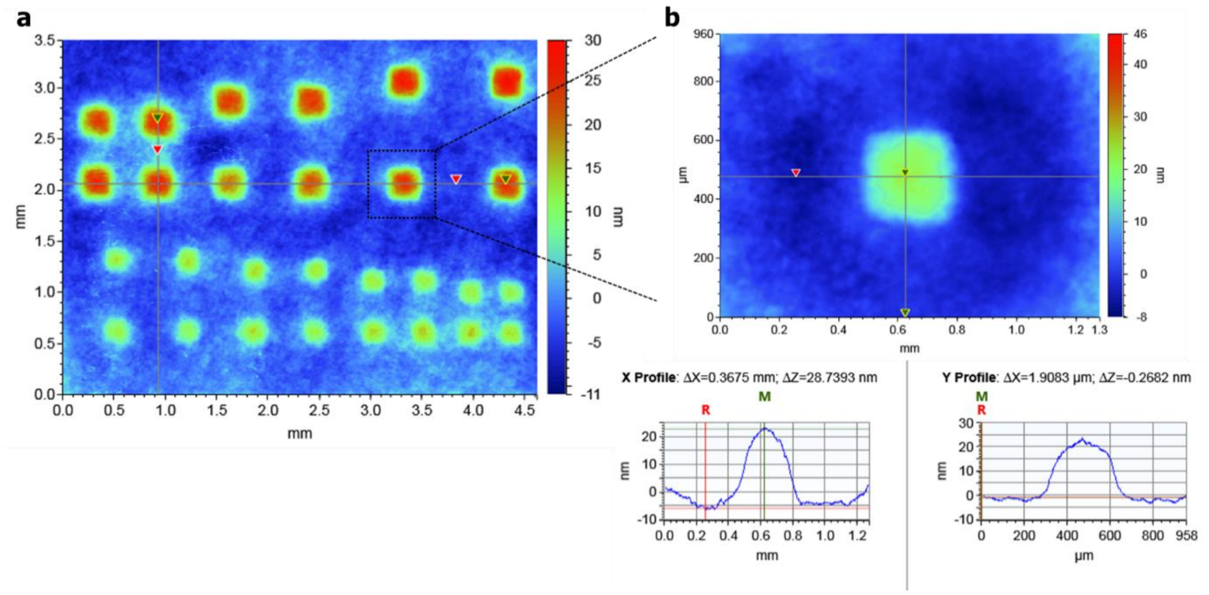

Supplementary Figure 27 | 3D-profile images of patterned PV3D3. a, 3D-profile image of PV3D3 spots with a thickness of ~30 nm. **b,** Magnified 3D-profile image.

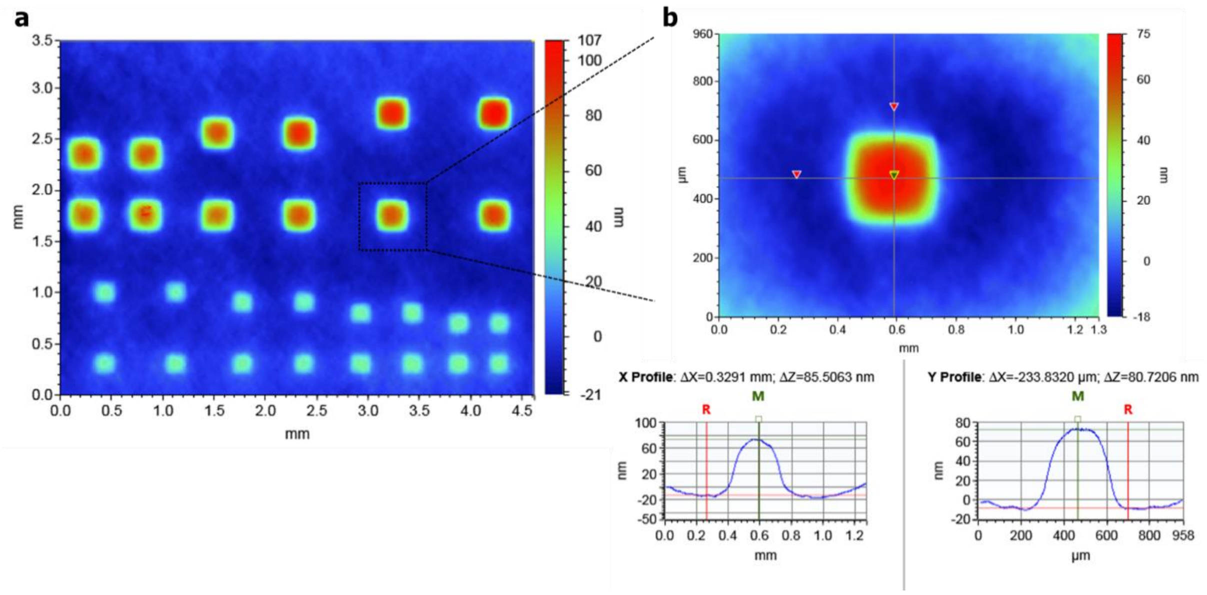

Supplementary Figure 28 | 3D-profile images of patterned PV3D3. a, 3D-profile image of PV3D3 spots with a thickness of ~80 nm. **b,** Magnified 3D-profile image.

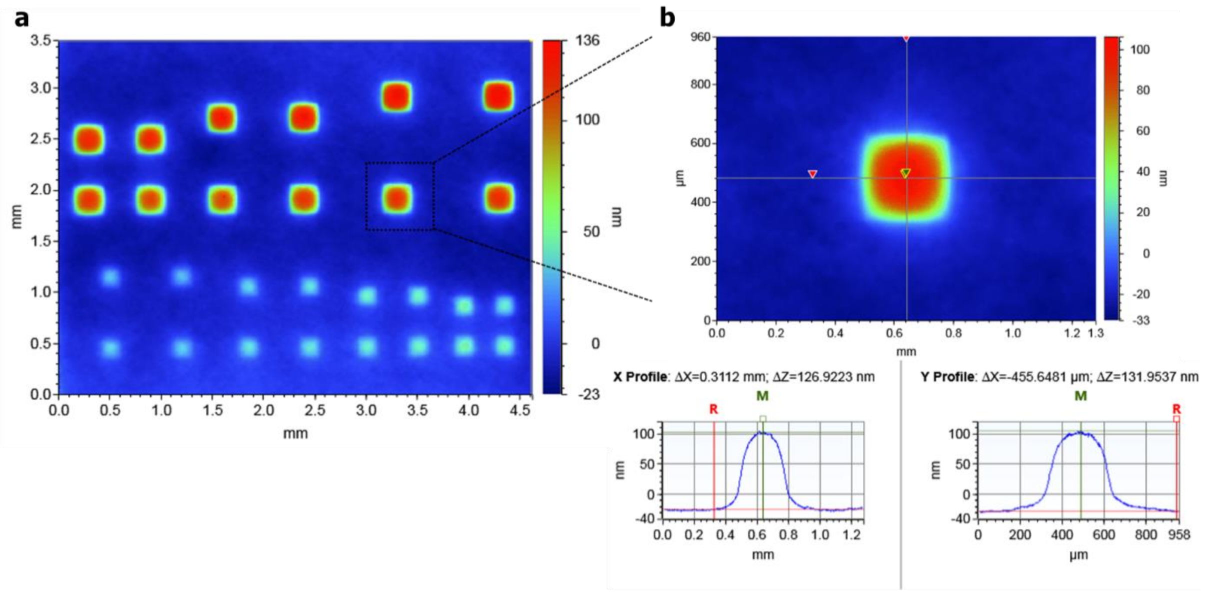

Supplementary Figure 29 | 3D-profile images of patterned PV3D3. a, 3D-profile image of PV3D3 spots with a thickness of ~130 nm. **b,** Magnified 3D-profile image.

Supplementary Tables

Supplementary Table 1 | Comparison of 3D-stacked transistors.

	Via-hole-less dielectric pattern method	Interconnect method not reported			Laser drilling	Inkjet printing
	This work	Ref. 12	Ref. 13	Ref. 17	Ref. 15	Ref. 16
Semiconductor	DNTT<br>PTCDI-C13	Pentacene<br>a-IGZO	TIPS-Pen<br>PCBM	F8BT<br>a-IGZO	Dif-TES-ADT<br>N2200	TIPS-Pen<br>N2200
Dielectric	PV3D3	Al_2O_3	Al_2O_3 /Cytop	Parylene	Parylene	PVP /Cytop
Operating voltage (V)	~10	~6	~8	~30	~20	~30
# of dielectric layers	~9	~2	~3	~2	~3	~3
# of stacked transistors	5	2	2	2	2	2
# of metal layers	20	3	3	3	~4	3

Supplementary Notes

Supplementary Note 1: Interfacial Trap Density

In 3D-OTFTs, the interfacial trap density (N_t) values were extracted using Equation (1):

$$N_t = \left[\frac{SS\ \log(e)}{kT/q} - 1\right] \cdot \frac{C_i}{q} \quad \text{where } SS \text{ is subthreshold swing} \tag{1}$$
